# *Sinosciapus* from Taiwan with description of a new species (Diptera, Dolichopodidae)

**DOI:** 10.3897/zookeys.159.2252

**Published:** 2011-12-23

**Authors:** Ding Yang, Yajun Zhu

**Affiliations:** 1 Department of Entomology, China Agricultural University, Beijing 100193, China; 2 Shanghai Entry-Exit Inspection and Quarantine Bureau, Shanghai 200135, China

**Keywords:** Diptera, Dolichopodidae, *Sinosciapus*, new species

## Abstract

*Sinosciapus liuae* **sp. n.** is newly described from Taiwan. The genus *Sinosciapus* is discussed and a key to the three known Oriental species is provided.

## Introduction

*Sinosciapus* Yang, 2001 is a small genus of the family Dolichopodidaewith only two previously known Oriental species from Southwest China and South China (Yang et al. 2006; Yang et al. 2011). The genus is characterized by the following features: eyes in both sexes narrowly separated; vertex in both sexes with strong vertical bristle; arista subapical, slightly shorter than width of head; lateral scutellar bristle strong, about 2/3 as long as apical one; fore tarsomere 5 with an elongated spine-like claw (Yang 2001; Yang et al. 2011).

In the present paper, the genus *Sinosciapus* is newly recorded from Taiwan with one new species, based on specimens collected by Ms. Xiaoyan Liu in Taiwan in 2011. An updated key to the three known species of *Sinosciapus* is given.

## Material and methods

The type specimens are deposited in the Entomological Museum of China Agricultural University, Beijing (CAU). The following abbreviations are used: acr = acrostichal, ad = anterodorsal, dc = dorsocentral, h = humeral, npl = notopleural, oc = ocellar, ph = posthumeral, psa = postalar, pvt = postvertical, sa = supraalar, su = sutural, sc = scutellar, vt = vertical.

## Taxonomy

### Genus Sinosciapus

Yang, 2001

http://species-id.net/wiki/Sinosciapus

Sinosciapus  Yang, 2001: 432. Type species: *Sinosciapus tianmushanus* Yang, 2001 (monotypy).

#### Diagnosis.

Eyes in both sexes narrowly separated, frons and face rather narrow (face less than 1/2 as wide as one eye, frons narrower than one eye). Vt strong. Clypeus apically separated from inner margin of eyes. Cheeks of females posteriorly with 1 black spine-like bristle. First flagellomere subtrapezoid or semicircular; arista subapical, slightly shorter than width of head. 5 strong dc, anterior 3 dc somewhat shorter; 5–7 irregularly paired acr very short and hair-like; scutellum with two pairs of strong sc, basal pair 2/3 as long as apical pair. Fore coxa with 3 bristles in male, with 3-4 spine-like bristles and many short spine-like bristles on inner surface in female. Wing: m-cu straight. Male genitalia rather small with well developed cercus like in *Condylostylus*.

#### Remarks.

*Sinosciapus* is an Asian genus, with three known species from subtropical or tropical forests of Southwest China and South China (Fig. 5). It is similar to *Amblypsilopus* in following points: arista rather short, shorter than head width; tibial chaetotaxy often weak, especially in males; m-cu usually straight. But it can be separated from the latter by the following features: eyes in both sexes narrowly separated, frons and face rather narrow (frons narrower than one eye); both sexes with strong vt; lateral sc strong, about 2/3 as long as apical one; fore tarsomere 5 with 1 elongated spine-like claw. In *Amblypsilopus*, the eyes in both sexes are widely separated, the frons and face are rather wide (frons wider than one eye), the male vertex has vt weak or lost; the lateral sc is always reduced to the weak hairs or lost; the claws on fore tarsomere 5 are normal (Bickel 1994; Yang et al. 2011).

#### Key to world species (males) of the genus *Sinosciapus*

**Table:** 

1	First flagellomere as long as wide; cercus weakly or distinctly curved; epandrium with apical margin not truncate in lateral view	2
–	First flagellomere longer than wide, trapezoid; cercus (except base) straight; epandrium with truncate apical margin in lateral view.	*Sinosciapus tianmushanus* Yang
2	Thorax and abdomen mostly metallic green; antenna blackish; first flagellomere semicircular; cercus distinctly curved; all tarsomeres 4-5 distinctly shortened; epandrium with lower apical corner acute in lateral view	*Sinosciapus yunlonganus* Yang & Saigusa
–	Thorax and abdomen mostly yellow; antenna yellow except first flagellomere dark yellow; first flagelloemre nearly trapezoid; fore tarsomere 4 shortened, mid tarsomere 5 shortened, and hind tarsomeres 4-5 shortened and thickened; cercus weakly curved; epandrium with upper apical corner acute in lateral view	*Sinosciapus liuae* sp. n.

### Sinosciapus tianmushanus

Yang, 2001

http://species-id.net/wiki/Sinosciapus_tianmushanus

Sinosciapus tianmushanus Yang, 2001: 432. Type locality: Zhejiang, Tianmushan.

#### Diagnosis.

Thorax and abdomen chiefly yellow. Antenna yellow. First flagellomere longer than wide, nearly trapezoid. Epandrium with truncate apical margin in lateral view; cercus (except base) straight; aedeagus with thickened apical portion long and paralleled. Fore tarsomere 4 not shortened, mid tarsomere 5 distinctly shortened, and hind tarsomere 4 slightly shortened.

#### Specimens examined.

Holotype ♂, Zhejiang, Tianmushan, Sanliting, 640 m, 30°26'N, 119°34'E, 1998. V. 30, Hong Wu (CAU). Paratypes 3♂♂3♀♀, same data as holotype (CAU).

#### Distribution.

Zhejiang.

#### Remarks.

This species distinctly differs from other two species of the genus in following features: first flagellomere longer than wide, trapezoid; cercus (except base) straight; epandrium with truncate apical margin in lateral view.

### Sinosciapus yunlonganus

Yang & Saigusa, 2001

http://species-id.net/wiki/Sinosciapus_yunlonganus

Sinosciapus yunlonganus Yang & Saigusa, 2001: 180. Type locality: Yunnan, Yunlong.
Amblypsilopus dirinus  Wei & Song, 2006: 310. Type locality: Guizhou, Chishui.

#### Diagnosis.

Thorax and abdomen mostly metallic green. Antenna blackish. First flagellomere as long as wide, semicircular. All legs with tarsomeres 4-5 distinctly shortened. Epandrium with lower apical corner acute in lateral view; cercus distinctly curved upward.

#### Specimens examined.

Holotype ♂, Yunnan, Yunlong, 24°52'N, 101°35'E, 1996. VI. 4, T. Saigusa (CAU). Paratype 1♂, Yunnan, Pingbian, Daweishan Mountain, 1800–2000 m, 22°58'N, 103°41'E, 1996. V. 24, T. Saigusa (CAU).

#### Distribution.

Yunnan, Guizhou.

#### Remarks.

This species is easily separated from *Sinosciapus tianmushanus* Yang by the thorax and abdomen being mostly metallic green, and first flagellomere as long as wide and semicircular.

### Sinosciapus liuae sp. n.

urn:lsid:zoobank.org:act:0BD7F6C1-012C-41A6-A25F-6FBF72937656

http://species-id.net/wiki/Sinosciapus_liuae

Figs 1–3 4 

#### Diagnosis.

Thorax and abdomen mostly yellow. Antenna yellow except first flagellomere dark yellow; first flagellomere as long as wide. Palpus and proboscis yellow. Legs yellow except tarsi brown to dark brown apically. Fore tarsomere 4 shortened, mid tarsomere 5 shortened, and hind tarsomeres 4-5 shortened and thickened.

#### Description.

Male. Body length 3.0–3.7 mm, wing length 3.6–3.7 mm.

Head metallic green except clypeus dark yellow, with pale gray pollen. Hairs and bristles on head black, except middle and lower postoculars (including postero-ventral hairs) pale yellow. Ocellar tubercle distinct, with pair of long oc and 2 very short posterior hairs. 1 vt on slope shorter than oc; 1 pvt at the end of postoculars row nearly as long as vt. Antenna yellow except first flagellomere dark yellow; first flagellomere nearly trapezoid in lateral view, as long as wide; arista subapical, blackish with blackish pubescence, shorter than head width. Palpus yellow, with pale hairs and 1 blackish apical bristle. Proboscis yellow, with short pale hairs.

Thorax mostly yellow with pale gray pollen, except pronotum pale metallic green medially, mesonotum mostly metallic green with large antero-lateral area including humerus and small postero-lateral area including postalar callus yellow, scutellum entirely metallic green, postnotum narrowly metallic green at middle. Hairs and bristles on thorax black. 5–6 short irregularly paired acr; 5 strong dc. 1 short hair-like h, 1 ph, 1 su, 2 sa, 1 psa, 2 npl; scutellum with pair of long apical sc and pair of short lateral sc (about 2/3 as long as apical sc). Legs yellow, but fore tarsomere 5 dark brown, mid tarsomeres 3–4 brown and tarsomere 5 dark brown, hind tarsomere 3 pale brown and tarsomeres 4–5 dark brown. Hairs and bristles on legs black. Fore coxa with 4 bristles apically; mid coxa with 3 bristles apically; hind coxa with 1 exterior bristle at basal 1/3. Fore tibia with 3 weak bristles only at tip; mid tibia with 1 short ad at base and 3 bristles at tip; hind tibia with 3 bristles only at tip. Fore tarsomere 4 shortened, tarsomere 5 with two rows of ventral spines and 1 long hook-like claw. Mid tarsomere 5 shortened. Hind tarsomeres 4–5 shortened and thickened. Relative length ratio of tibiae and tarsomeres: LI 5.5 : 4.0 : 1.2 : 0.9 : 0.4 : 0.8; LII 7.7 : 5.9 : 1.3 : 1.2 : 0.8 : 0.35; LIII 11.1 : 5.1 : 1.6 : 1.0 : 0.4 : 0.6. Wing hyaline, slightly tinged with brownish, veins dark brown. Vein M_1_ with strongly curved basal portion nearly geniculate, vein M_2_ reduced but only visible at base. Crossvein m-cu nearly straight. Squama dark yellow, with long brown hairs. Halter dark yellow.

Abdomen partly metallic green with pale gray pollen, except tergites 2–5 with anterior and lateral portions yellow, sternites 1–5 yellow except posterior margin of sternite 5 brown. Hairs and bristles on abdomen black and weak, but hairs on sternites 1–5 pale yellow. Hypopygium black except cerci and hypandrium brownish.

Male genitalia (Figs. 2–3). Epandrium somewhat quadrate, nearly as long as wide, and with oblique apical margin and upper apical corner acute in lateral view. Surstylus short and wide with 2 long bristles. Cercus very long, about 4 times as long as epandrium, basally very thick, apically long finger-like, slightly curved. Hypandrium long conical. Phallus with short thick apical portion slightly narrowed toward extreme tip in ventral view.

Female. Body length 3.3–5.0 mm, wing length 3.6–4.4 mm. Similar to male, but different in following points: mesonotum yellow with a large metallic green mid-posterior spot nearly triangular and two small pale metallic green lateral spots nearly semicircular. Mid coxa with a brown outer stripe. Fore coxa with 4 long spine-like apical bristles and several very short spine-like anterior bristles. Abdominal tergite 1 also yellow except posterior margin metallic green. Legs with only tarsomere 5 short. Relative length ratio of tibiae and tarsomeres: LI 5.8 : 5.1 : 1.7 : 1.2 : 0.8 : 0.55; LII 7.9 : 6.8 : 1.35 : 1.2 : 0.8 : 0.4; LIII 11.1 : 5.2 : 1.75 : 1.2 : 0.8 : 0.4.

**Figures 1–3.**
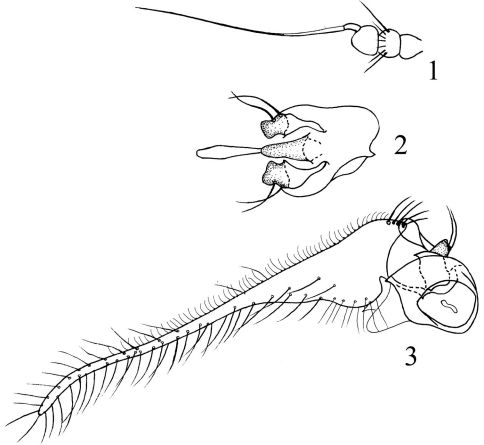
*Sinosciapus liuae* sp. n. (male) **1** antenna, lateral view **2** genitalia excluding cerci, ventral view **3** genitalia, lateral view.

**Figure 4.**
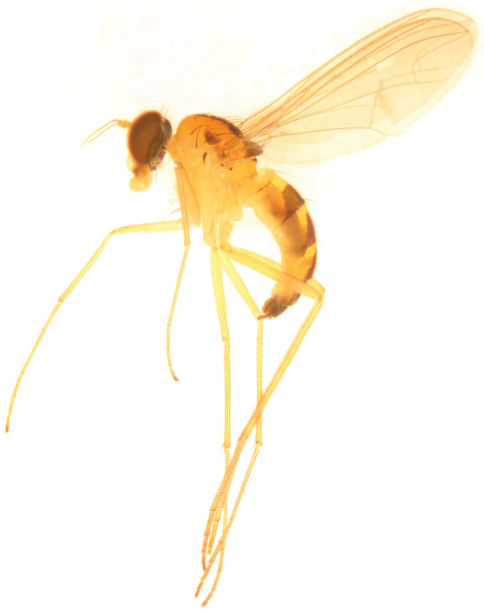
*Sinosciapus liuae* sp. n. (female).

**Figure 5.**
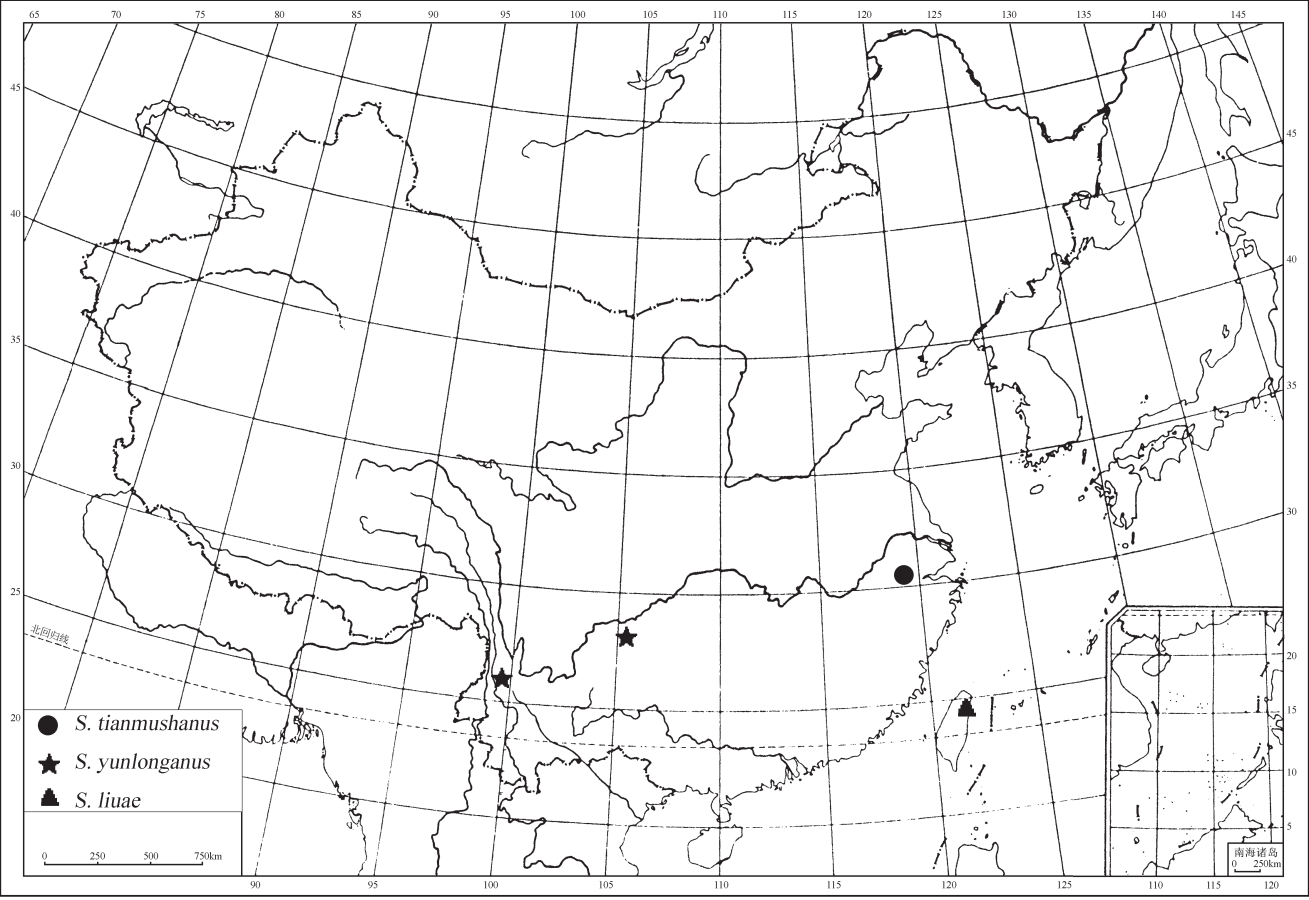
Distribution of *Sinosciapus*.

#### Type material.

Holotype ♂, Taiwan, Ilan County, Mingchin Forest Recreation Area, 1130 m, 24°45'N, 121°44'E, 2011. VI. 13, Xiaoyan Liu (CAU). Paratypes 1♂10♀♀, same data as holotype (CAU); 1♂3♀♀, Taiwan, Hualien County, Pilu, Shenmu, 2150 m, 23°59'N, 121°36'E, 2011. VI. 20, Xiaoyan Liu (CAU).

#### Distribution.

Taiwan.

#### Etymology.

The specific name refers to the collector Ms. Xiaoyan Liu.

#### Remarks.

The new species is somewhat similar to *Sinosciapus yunlonganus*Yang *et* Saigusa, 2001 in having the first flagellomere as long as wide and epandrium with the oblique apical margin in lateral view. But it can be separated from the latter in the following features: thorax and abdomen mostly yellow, first flagelloemre nearly trapezoid, cercus weakly curved, epandrium with upper apical corner acute in lateral view; in *Sinosciapus yunlonganus*, thorax and abdomen mostly metallic green, first flagellomere semicircular, cercus distinctly curved, epandrium with lower apical corner acute in lateral view (Yang & Saigusa 2001; Yang et al. 2001).

## Supplementary Material

XML Treatment for Sinosciapus

XML Treatment for Sinosciapus tianmushanus

XML Treatment for Sinosciapus yunlonganus

XML Treatment for Sinosciapus liuae
